# PTHrP on MCF-7 breast cancer cells: a growth factor or an antimitogenic peptide?

**DOI:** 10.1038/sj.bjc.6601689

**Published:** 2004-03-16

**Authors:** E Maioli, V Fortino

**Affiliations:** 1 Department of Physiology, Via Aldo Moro, Siena 8-53100, Italy

**Sir**,

We read with interest the publication by Hoey *et al* (2003), on MCF-7 cell proliferation promoted by the overexpressed PTH/PTHrP receptor, since a study on the effects of PTHrP on MCF-7 cell proliferation is currently in progress in our Lab.

We knew that the old and new literature reports contradictory findings in this cancer cell line as far as the proliferative response is concerned; it is surprising, however, that the question is still open in 2003. To our knowledge, the first study on PTHrP and MCF-7 cells is the one by Birch *et al* (1995), cited in Hoey's paper. However, discordant results had already been presented in 1981 (Cho-Chung *et al*, 1981). Birch's group found a mitogenic effect of PTH and PTHrP on MCF-7 cells, with a parallel increase in cAMP intracellular levels. Therefore, the work by Hoey *et al* (2003) appears to support Birch's findings.

Conversely, more recent studies, also cited in the paper, show that the cAMP pathway inhibits proliferation in MCF-7 cells (Chen *et al*, 1998).

The recent report by Tovar Sepulveda *et al* (2002), focused on PTHrP and MCF-7, is particularly instructive here, although it does not meet the question of the different response to PTHrP by the same cell. They clearly demonstrated that PTHrP possesses a double signalling on MCF-7: an antimitogenic pathway mediated by the membrane receptor and a second wave of signalling, pro-proliferative and antiapoptotic, based on nuclear localisation of the peptide. The same dual effect had also been described in vascular smooth muscle cells in 1997 (Massfelder *et al*, 1997).

We believe that this controversial aspect is not discussed enough in Hoey's article. However, we can realise that in May 2002, the date of the first submission, the author could not have read the paper by Tovar Sepulveda *et al* (2002). On the contrary, Hoey knew and cited the work by Falzon and Du (2000). He suspects that Falzon's results about the intracrine effect of PTHrP in PTHrP-transfected MCF-7 cells is of poor physiological relevance, since it is unlikely that the same phenomenon takes place in parental cells as well.

We agree that transfection, in general, may confer cell characteristics completely different from those of the parental one and this is the main risk in transfection experiments. However, the same remark could be made to Hoey's results, too.

Our preliminary results, summarised in Figure 1

**Figure 1:**
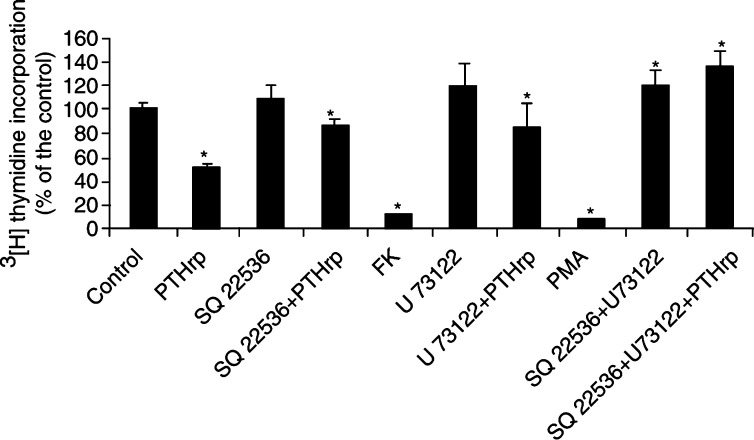
Proliferative response of MCF-7 breast cancer cells to 640 nM PTHrp 1–40. Cells were grown in MEM in the presence of 2.5% FCS. Chemical inhibitors and activators were used at the following doses: SQ 22536: 50 *μ*M; Forskolin (FK): 100 *μ*M; U 73122: 20 *μ*M; PMA: 0.3 *μ*M. Results are expressed as % of the control. Data are the mean value±s.d. of two independent experiments, each performed in duplicate.^*^Significantly different from the control (*P*<0.05).

, strongly differ from Hoey's ones. In fact, our MCF-7 cells respond to 640 nM PTHrP, in contrast to Hoey's cells, which are unresponsive to 125 nM PTHrP.

This is the first difference with Hoey's work.

On working with parental MCF-7 cells, we needed to increase the PTHrP dose to obtain some effect on proliferation. We also failed to appreciate significant variations below the dose of 640 nM. Thus, MCF-7 cells are not unresponsive to PTHrP; they simply need a stronger treatment.

Our results (still incomplete to be collected in a manuscript) also indicate that exogenous PTHrP triggers two main transduction pathways: the adenylyl-cyclase (AC)/protein kinase (PK) A and the phospholipase (PL) C/PKC cascade. Thus, the native PTH/PTHrP receptor is coupled also to the Ca^2+^ signalling route in MCF-7 cells.

This is the second difference: Hoey's cells have probably lost this transduction pathway.

In our MCF-7 cells, both PKA and PKC are antimitogenic and this appears to be the main difference. We cannot tell, at the moment, which is the major player, but the selective block of AC or PLC reduces the antiproliferative effect of PTHrP.

We chose to block the membrane enzymes, rather than PKs, because, as reported also by Hoey, chemical inhibitors of PKs are toxic for cells and mask the proliferative effect of a test factor.

This is the only experience we have in common with Hoey's group.

Hoey describes an effect of PTHrP on cell sensitivity to growth factors, but this is not a novel phenomenon, as it was already described by Linseman *et al* (1995). Probably, Hoey is dealing with the so-called transactivation of growth factors tyrosine kinase (TK) receptors by PTHrP G-protein-coupled receptor (GPCR) (Linseman *et al*, 1995; Lowes *et al*, 2002). We observed a similar phenomenon in 2002 while culturing skin fibroblasts in the presence of serum (then of growth factors) (Maioli *et al*, 2002).

We do not have the presumption to solve the conflict between Hoey's and our results, but in an attempt to reconcile them, we hypothesise that the overexpressed receptor, unable to couple to PLC, thus lacking one of the two antiproliferative pathways, acquired enhanced ability to transactivate TK receptors; the pro-mitogenic effect predominates in the presence of 2% serum.

We believe that such a pro-proliferative action of PTHrP, mediated by the membrane receptor, is operative also in parental MCF-7 cells. In fact, in our preliminary study, exogenous 1–40 PTHrP shares pro-mitogenic properties in 2.5% serum-cultured MCF-7 cells, when the membrane AC and PLC are simultaneously blocked.

Finally, the main question and the only one that cannot be ascribed to the transfection process is the opposite effect of forskolin (an AC activator) on parental (ours) and transfected MCF-7 cells (Hoey's ones). In fact, it is now clear that cAMP can exert both pro- and antimitogenic effects acting at the level of different Raf isoforms. As a rule, the cAMP/PKA pathway stimulates Ras-independent and Rap-1-dependent extracellular regulated kinase (ERK) phosphorylation and cell proliferation in Raf-B-expressing cells, but it inhibits growth in Raf-B-negative cells (Fujita *et al*, 2002).

Then, is the difference in cAMP levels sufficient to explain the discrepancy?

Our previous experience on skin fibroblasts, which express predominantly the Raf-1 isoform (Fortino *et al*, 2002), indicates that forskolin (then cAMP) is always antiproliferative, at any dose tested (Maioli *et al*, 2002). Consistently, in our MCF-7 cells, forskolin (as well as phorbol 12-myristyl 13-acetate, PMA) mimics the PTHrP antimitogenic effect. Although a study focused on Raf isoforms in MCF-7 cells is still missing, Raf-1 isoform is surely expressed (Weinstein-Oppenheimer *et al*, 2001).

Clarifying this and the other aspects would add significantly to our present biological and clinical knowledge on PTHrP role and responsibility in cancer.

## References

[bib1] Birch MA, Carron JA, Scott M, Fraser WD, Gallagher JA (1995) Parathyroid hormone (PTH)/PTH-related protein (PTHrP) receptor expression and mitogenic responses in human breast cancer cell lines. Br J Cancer 72: 90–95 7599071 10.1038/bjc.1995.282 PMC2034135

[bib2] Chen J, Bander JA, Santore TA, Chen Y, Ram PT, Smit MJ, Iyengar R (1998) Expression of Q227L-Gα_s_ in MCF-7 human breast cancer cells inhibits tumorigenesis. Proc Natl Acad Sci USA 95: 2648–2652 9482941 10.1073/pnas.95.5.2648 PMC19449

[bib3] Cho-Chung YS, Clair T, Bodwin JS, Berghoffer B (1981) Growth arrest and morphological change of human breast cancer cells by dibutyryl cyclic AMP and L-arginine. Science 214: 77–79 6269181 10.1126/science.6269181

[bib4] Falzon M, Du P (2000) Enhanced growth of MCF-7 breast cancer cells overexpressing parathyroid hormone-related peptide. Endocrinology 141: 1882–1892 10803599 10.1210/endo.141.5.7470

[bib5] Fortino V, Torricelli C, Gardi C, Valacchi G, Rossi Paccani S, Maioli E (2002) Erks are the point of divergence of PKA and PKC activation by PTHrP in human skin fibroblasts. Cell Mol Life Sci 59: 2165–2171 12568342 10.1007/s000180200015 PMC11146128

[bib6] Fujita T, Meguro T, Fukuyama R, Nakamuta H, Koida M (2002) New signaling pathway for parathyroid hormone and cyclic AMP action on extracellular-regulated kinase and cell proliferation in bone cells. Checkpoint of modulation by cyclic AMP. J Biol Chem 277: 22191–22200 11956184 10.1074/jbc.M110364200

[bib7] Hoey RP, Sanderson C, Iddon J, Brady G, Bundred NJ, Anderson NG (2003) The parathyroid hormone-related protein receptor is expressed in breast cancer bone metastases and promotes autocrine proliferation in breast carcinoma cells. Br J Cancer 88: 567–573 12592371 10.1038/sj.bjc.6600757 PMC2377170

[bib8] Linseman DA, Benjamin CW, Jones DA (1995) Convergence of angiotensin II and platelet-derived growth factor receptor signaling cascades in vascular smooth muscle cells. J Biol Chem 270: 12563–12568 7759503 10.1074/jbc.270.21.12563

[bib9] Lowes VL, Ip NY, Wong YH (2002) Integration of signals from receptor tyrosine kinases and G protein-coupled receptors. Neurosignals 11: 5–19 11943878 10.1159/000057317

[bib10] Maioli E, Fortino V, Torricelli C, Arezzini B, Gardi C (2002) Effect of parathyroid hormone-related protein on fibroblast proliferation and collagen metabolism in human skin. Exp Dermatol 11: 302–310 12190938 10.1034/j.1600-0625.2002.110403.x

[bib11] Massfelder T, Dann P, Wu TL, Vasavada R, Helwig JJ, Stewart AF (1997) Opposing mitogenic and anti-mitogenic actions of parathyroid hormone-related protein in vascular smooth muscle cells: a critical role for nuclear targeting. Proc Natl Acad Sci USA 94: 13630–13635 9391077 10.1073/pnas.94.25.13630 PMC28357

[bib12] Tovar Sepulveda VA, Shen X, Falzon M (2002) Intracrine PTHrP protects against serum starvation-induced apoptosis and regulates the cell cycle in MCF-7 breast cancer cells. Endocrinology 143: 596–606 11796515 10.1210/endo.143.2.8645

[bib13] Weinstein-Oppenheimer CR, Henriquez-Roldan CF, Davis JM, Navolanic PM, Saleh OA, Steelman LS, Franklin RA, Robinson PJ, McMahon M, McCubrey JA (2001) Role of the Raf signal transduction cascade in the *in vitro* resistance to the anticancer drug doxorubicin. Clin Cancer Res 7: 2898–2907 11555608

